# The Femoral Nerve Block Setting the Agenda for Nursing Care of Older Patients With hip Fractures—A Qualitative Study

**DOI:** 10.1177/23779608231177533

**Published:** 2023-05-30

**Authors:** Anna Unneby, Birgitta Olofsson, Britt-Marie Lindgren

**Affiliations:** 1 375281 Department of Nursing, Umeå University, Umeå, Sweden; 2 206100 Department of Surgical and Perioperative Science Orthopedics, Umeå University, Umeå, Sweden

**Keywords:** experiences, femoral nerve block, hip fracture, nursing care, qualitative content analysis

## Abstract

**Introduction:**

Hip fractures among older people are common worldwide, and it is often associated with preoperative pain. Due to increased comorbidity and high age, traditional pain relief can be a challenge. An alternative to traditional pain relief is a femoral nerve block, which is safe and suitable for anesthesia and analgesia for hip fractures among patients with or without dementia. It is essential to provide adequate pain management, and nurses report negative attitudes toward opioids and seem to prefer alternative pain management. To our knowledge, no study has focused on staff's experiences of nursing care for patients treated with femoral nerve block.

**Aim:**

To describe staff's experiences providing nursing care in preoperative pain and pain management to older patients with a hip fracture who received a femoral nerve block.

**Design:**

A qualitative exploratory design.

**Method:**

Semistructured interviews with 19 nurses or assistant nurses in an orthopedic ward or emergency department. They were experienced in caring for patients with hip fractures who received treatment with a femoral nerve block. The interviews were subjected to qualitative content analysis.

**Results:**

Staff described the femoral nerve block as setting the agenda when caring for older patients with hip fractures in the preoperative phase. The outcome of the femoral nerve block affected nursing care, depending on if the femoral nerve block was successful or not. Nursing care requires timing, with a need for staff orienting to time and customizing their communication. Further, staff faced ethical challenges regarding doing good and not harm, relieving pain, and avoiding side effects.

**Conclusions:**

The femoral nerve block was an important issue for nursing staff in patients with hip fractures in the preoperative phase. Our results point toward the benefits of giving femoral nerve blocks as soon as possible to facilitate nursing care, however, this should be studied in future research.

## Introduction

Hip fractures are common, and the Nordic countries have the highest incidence rate worldwide (Kanis et al., 2012). However, the incidence rate has declined over the years among the Swedish population. Patients with hip fractures are vulnerable to increasing comorbidity with a high mortality risk, disability, and pain (Bano et al., 2020; Ceolin et al., 2023; Elsevier & Cannada, 2020; Meyer et al., 2021).

### Literature Review

Hip fractures are associated with preoperative pain (Cowan et al., 2017), and due to increased comorbidity and high age, traditional pain relief can often be a challenge (Elsevier & Cannada, 2020). An alternative is a femoral nerve block (FNB). FNB is suitable for anesthesia and analgesia for hip fractures among patients with or without dementia. Further, FNBs are safe, with no reported adverse events in the studies included in the review from Riddell et al. (2016). FNB has been shown to reduce pain and the use of systemic analgesia for patients with hip fracture compared to those only receiving systemic analgesia; this is also true among those with dementia (Skjold et al., 2020; Unneby et al., 2017). Studies have shown that 46%–50% of patients with a hip fracture have a dementia disorder (Karlsson et al., 2020; Unneby et al., 2020), and patients with dementia have an increased risk of sustaining a hip fracture (Hou et al., 2021). Moreover, a review showed that patients with dementia after a hip fracture receive fewer opioid analgesics than cognitively intact patients (Moschinski et al., 2017).

For a nurse, it is essential to provide adequate pain management (Biz et al., 2019). Nurses are responsible for administering analgesia, assessing, and evaluating effects and side effects (Jensen et al., 2018). Research shows that nurses report negative attitudes toward opioids. Reasons reported for this could be a lack of confidence and knowledge of using opioids and fear of legal persecution by surgical patients. Particularly nurses with more extended experience seem to prefer alternative pain management (Shoqirat et al., 2019). A study from Unneby et al. (2022) showed that patients suggested limiting opioids and that FNB is an excellent alternative if opioids cause side effects.

One study showed misunderstanding regarding pain between staff and patients (Ivarsson et al., 2018). The patients in that study described that sometimes they experienced severe pain while other times they were adequately treated. On some occasions, the staff were unsure how much to give, and sometimes they gave painkillers even though the patients did not want painkillers. This resulted in great remorse and discomfort for the patients. Among patients with dementia, it is a challenge to differentiate behavioral expressions and pain (Gagliese et al., 2018). Communication regarding pain is described as challenging when patients develop delirium or are cognitively impaired (Ivarsson et al., 2018). Patients had described how FNB made them feel relaxed and relied on the analgesic effect after receiving an FNB, causing them not to feel pain when staff performed nursing actions (Unneby et al., 2022).

To the best of our knowledge, no study has focused on staff's experiences of nursing care for patients treated with FNB. In a small study by Kullenberg et al., (2004) it was discussed that nurses’ experiences of care could be facilitated if patients were treated with FNB. Therefore, this study aims to describe the staff's experiences providing nursing care in preoperative pain and pain management to older patients with hip fractures who received an FNB.

## Method

### Design

We used a qualitative exploratory design because of its strength in studying phenomena in a naturalistic context (Sandelowski, 2000).

### Context/Setting

The study was conducted at one hospital with staff in an orthopedic ward (OW) or emergency department (ED). In this paper, registered and assistant nurses are named collectively as staff. The anesthesiologist is the one performing the FNB using ultrasound guidance. Whether patients received FNB at OW or ED depended on whether the anesthesiologist had time and if the staff at the ED called for an FNB. Otherwise, a nurse in OW called the anesthesiologist, informing them of the need for FNB for a patient with a hip fracture.

### Participants

A purposive sample of nursing staff who could narrate experiences of pain and pain management among patients with hip fractures who had received an FNB was used to provide suitable variation for this study. Staff in the OW and ED were informed about the study in workplace meetings and short morning meetings called the “daily meeting.” The inclusion criteria were nurses or assistant nurses in the OW or ED with experience caring for patients with hip fractures who received treatment with FNB and could speak and understand Swedish. Interested participants contacted the first author and informed if they wanted to participate; contact took place at work, via email, or on social media through a private message. All participants eligible for inclusion were given verbal and written information about the study by the first author. Those agreeing signed a consent form, and the time and place chosen by the participant were set for the interview. Background characteristics were collected through a questionnaire about gender, age, professional degree, and years of experience at the specific ward.

### Data Collection

The first author conducted all individual interviews except one, which a trained Ph.D. colleague conducted. The interview guide was semi-structured, and the same for the staff, independent of which ward the participant worked in and any professional degree. However, the specific questions about the experience of giving opioids were excluded from the interviews with the assistant nurses. Examples of questions from the interview guide are: Can you tell me about the preoperative care among patients with a hip fracture? Can you tell me if you experience any difference among the patients before and after those with a hip fracture receive an FNB? How do you feel as a nurse or assistant nurse when the patient has to wait a long time for an FNB? Can you tell me when patients with dementia disorder receive an FNB? The interviews were conducted between January 2018 and February 2020. The interviews lasted between 15 and 38 min (median = 22 min) and were transcribed verbatim.

### Data Analysis

Qualitative content analysis was used to analyse the interviews (Graneheim & Lundman, 2004; Graneheim et al., 2017; Lindgren et al., 2020). All interviews were conducted before the analysis started. The first author read the transcribed interviews several times to become familiar with the data and to understand and gain a sense of the whole. To ensure trustworthiness, all authors read several of the transcribed interviews. The transcripts were imported into MAXQDA, a qualitative data management software program, to systemically sort and code the data (Kuckartz & Rädiker, 2019).

Next, the first author started the process of de-contextualization by dividing the interview texts into meaning units (words, sentences, or paragraphs related to the same central meaning). After that, the meaning units were condensed and labeled with codes describing the manifest content. Before the next step in the analysis, all authors took part in the coding before the recontextualization process, sorting those regarding similarities and differences. One example of the analysis process is that codes such as; “goes better with FNB, they allow more movement,” “with FNB they can sit up in bed,” “big difference with the block, and patients are not afraid anymore” were interpreted and sorted into the subcategory successful FNB.

All authors were involved in the analysis process, which led to an agreement on the final wording of subcategories, categories, and the main category.

## Results

In total, 19 nurses and assistant nurses were interviewed: 12 were registered nurses, and 7 were assistant nurses, aged between 24 and 57 years (median  =  38 years). They had between 6 months and 25 years of working experience (median  =  4 years). All of them were women except for one assistant nurse. Four of the registered nurses and assistant nurses worked in the ED.

Participants’ descriptions of their nursing care experiences in the preoperative pain and pain management of older patients with a hip fracture who received an FNB were divided into one main category, three categories, and six subcategories. Table 1 shows an overview of the results.

**Table 1.** Overview of the Results Revealed in the Analysis.

Subcategories	Categories	Main category
Orient to time Customise communication	Requires timing	*Femoral nerve block (FNB) setting the agenda*
Successful FNB Unsuccessful FNB	FNB outcomes affect nursing care	
Do good and not harm Relieve pain and avoid side-effects	Ethical challenges	

### FNB Setting the Agenda

Participants described that the FNB set the agenda when caring for older patients with hip fractures in the preoperative phase. Nursing care requires timing, where staff needs to orient time and customize their communication. The outcome of the FNB affects nursing care, depending on whether the FNB was successful or unsuccessful. Further, staff faces ethical challenges regarding doing good and not harm, relieving pain, and avoiding side effects.

#### Requires Timing

The FNB means that nursing care requires timing, with staff needing to orient to time depending on when FNB was given and customize their communication.

##### Orient to Time

The participants expressed how the work was adjusted depending on when the FNB was given. The waiting time for an FNB was described as a never-ending story. Participants emphasized that as a barrier in the work shift, claiming that it negatively affected the possibilities of caring for patients with a hip fracture. As one participant said about the waiting time for the FNB:

you want it to go fast, and it can be frustrating because it requires more of us in terms of nursing. Also, as long as they did not receive the FNB, they had more pain and were more insecure (P 15)

The participants described that sometimes, to be ready for surgery; they were forced to decide about performing the surgery shower before patients received the FNB. As one participant explained:

it does not feel good from a care perspective, and at the same time, when things have to be done at short notice, and we need to perform the surgery shower, there may not be time to wait for the FNB and then you have to make the best of the situation both for the patient and us (P 17)

However, participants expressed that even if there was enough staff to make it possible to perform the surgery shower, they decided to wait for the anesthesiologist to arrive and administer the FNB to facilitate and ensure good nursing actions. The participants also expressed the risk that complications remarkably increased if they had to wait too long for the FNB to perform nursing actions. Further, feelings of not doing their job as expected were described by participants when they had no time for surgery showering since they had prioritized waiting for an FNB. Some participants described wanting to apologize to staff working in the operation unit when patients were not prepared as expected.

Some participants mentioned that no evaluation from assistant nurses was performed after the patients received an FNB to assess whether it had any effect before starting with the surgery shower. One reason for this was that it was a stressful situation, and they wanted to be done with the surgery shower before the next shift started. Some participants also expressed that the assistant nurses need to gain knowledge about how the FNB worked and expected an effect. One participant said:

I think that if you, as a nurse or other colleagues, can assess the effect of the FNB and if you notice that the FNB has been successfully given, you are calmer as yourself when you perform movements with the patient (P 6)

Participants said that the FNB facilitated work if it was performed as soon as possible and that it should be a part of the admission routine in the ED. When patients arrived having been given an FNB at the ED, it improved flow in the preoperative phase, as staff described how they could start at once with the preparations for surgery. As one participant explained:

I can immediately start with a surgery shower, prepare and everything is ready for surgery, and for the patient, it must mean a lot because it is many movements, with turning the patient to check for decubitus ulcers (P 2).

##### Customise Communication

The participants were adamant that they needed to individualize communication according to how well the patients understood the purpose of the block and the procedure around it. Participants described how some patients understood quickly and felt calm, while others needed staff to be present to explain the block. Participants also said that it differed depending on which anesthesiologist performed the FNB. Some did it quickly and did not take the time to inform the patient about the block.

Not all anaesthesiologists are so informative, and sometimes they start pulling off the blanket at the same time as they explain the procedure or pull down patients’ panties without explanation, and then they start the procedure. It feels like not all patients are involved in what is happening (P 10)

Participants emphasized that the information could affect the result of the FNB and that not much was required for the patient to feel calmer regarding communication. It was also mentioned that some anesthesiologists only saw a “piece of meat” lying there who would receive an FNB. As one participant explained:

if the anaesthesiologist also calmly explains what to do, when to do it, and how to do it, well-informed patients also get better pain relief (P 2)

Participants made it clear that nurses had a central part in the communication. It was described how nurses discussed the plan for analgesics with the next of kin. Nurses were also responsible for communicating with the anesthesiologist about the need and time for an FNB and communicating with the assistant nurses about evaluating the block and timing about when to start the surgical shower.

The participants expressed that they needed to be more present by, for example, holding hands and finding distractions, such as talking about things, when caring for patients with dementia yet still informing the patients about the block and procedure. Further, they expressed that the distraction needed to be calm; otherwise, it would have the opposite effect. The participants described different aspects of communication with the anesthesiologist about patients with dementia. They said it was positive when they and the anesthesiologist communicated and worked together. This situation changed how successfully the block was administered to patients with hip fractures and dementia.

Participants said that when the anesthesiologist needed to interrupt and go in and out of the room, it sometimes made the patient with dementia agitated and insecure. Further, they emphasized how the anesthesiologist played an important role in determining how well it worked to give the block to patients with dementia. They described the patients as calmer if the anesthesiologist had a calm appearance, took their time, and individualized the meeting.

#### FNB Outcomes Affect Nursing Care

The outcome of the FNB affected nursing care depending on whether it was successful or unsuccessful.

##### Successful FNB

Participants described feeling calmer knowing that the FNB had decreased patients’ pain. They emphasized that it was possible to do a better job as all nursing actions became more manageable after a successful FNB.

I think that everyone experiences it so positively. Regarding nursing care, everyone experiences it as a relief, we are here for the patients, and that is why I think it is so fantastic because the patients do not have to lie down and have such terrible pain. I think it's so good with the block (P 15)

Participants said patients were not afraid of pain after a successful FNB and surrendered to and trusted the staff, making nursing actions easier. Participants expressed that when the FNB was successful, it became easier to move patients without causing suffering and increasing pain.

if patients come with a nerve block or get a nerve block, we can move the patient in a much better way, we can do a proper surgery-shower, and it reduces the suffering for the patient (P 17)

Further, raising the patient to a sitting position in the bed became more manageable, making it possible for the patient to eat if the fasting was interrupted. Some participants expressed that a significant part of a successful FNB was that nurses did not have the same need to be present multiple times as when opioids were given. They insisted that the FNB did not give patients any side effects and that they were less delirious than when they received opioids. However, some participants expressed that nursing actions can be done without FNB and stated there was a time before FNB was used for pain relief.

Participants explained that the patients with dementia became calmer and more relaxed when successfully given an FNB, making care more manageable. However, they also said that it included challenges and adjustments. Here they described that when the hip pain disappeared, the patients forgot about their hip fracture and wanted to stand up since the pain was no longer an obstacle. The staff needed to be more present here, which resulted in even more things to control. Participants described, on the one hand, the relief that patients with dementia were no longer in pain. On the other hand, it was considered a stressful situation since the staff could only sometimes be present with the patient. As one participant explained:

Sometimes, when a patient with dementia who received an FNB is anxious, we have to spare one assistant nurse to be with the patient. Unfortunately, we do not always have those resources, and that looks so damn bad (P 15)

Some participants reasoned whether it was better to avoid giving an FNB, allowing the patients to remain in pain since it reminded the patients with dementia not to move.

It's good that they get a block, of course, but it's bisected since patients with severe dementia still climb, and absolutely when they received the block. I don't know how often I have been with the patient because they climb over the bed since they don't feel that the hip is broken after receiving the block. [The interviewer asks a probing question] It is bisected; they are worried because they are in pain and want to move. It is a difficult balancing act [The interviewer asks a probing question] It depends on which patient it is, the patient last week, I had preferred that she did not receive the block, she climbed over everything just going bananas (P 16)

##### Unsuccessful FNB

Some participants expressed that they sometimes needed to be more careful when moving due to patients’ increased pain in the case of an unsuccessful FNB. Participants emphasized that it was challenging to help patients adjust movements when the block was unsuccessful. Depending on the FNB's success, they needed to adjust the situation and activities with the patient. As one participant said:

I feel there is not much I can do. I try to perform small movements, like a light blanket somewhere, so it will not be so heavy on the leg. I feel pretty bad because I feel that I cannot do my best with the patient (P 13)

Participants claimed that sometimes, they needed to step away from the routine of a surgery shower on a stretcher and instead wash the patient in bed. Some other participants said that even when an FNB was unsuccessfully, it was still possible to perform the surgery shower. However, some participants asserted it was more the pain intensity before the block that controlled the pain after the FNB rather than the success of a given FNB. The FNB's success depended on how patients experienced pain, which made the staff adjust to the situation. Participants considered it a barrier in nursing care if patients with dementia did not receive an FNB. As one participant exclaimed when the surgery shower needed to be performed without the patients with dementia receiving an FNB:

a patient with dementia with a hip fracture without an FNB is like rinsing over a glass instead of washing it (P 13).

#### Ethical Challenges

The FNB confronted staff with ethical challenges regarding doing good and not harm. Also, it challenged the practice of relieving pain and avoiding side effects.

##### Do Good and not Harm

Participants expressed that if they decided to give opioids, it was seen as an emergency solution. It was described as having passed a line in thinking that ensured it would be good.

I feel there is nothing that would be a good alternative instead of the FNB because it is no other treatment that gives well-relived pain, so it is no other pain treatment that you feel that is so fun to give (P 11)

it is an active waiting on FNB. People experience that it has such a great effect on the pain that it is not even worth to do anything before they receive the block (P 1).

Some participants described helping as difficult when patients did not receive an FNB since they were in pain and panicked before staff touched them. However, some other participants said that the experience of administering intravenous opioids was not stressful. They insisted that deciding to perform a surgery shower without patients having received an FNB made them feel that they were providing poor nursing care.

Participants expressed that sometimes colleagues needed to hold the patients with dementia when the FNB was given. On the one hand, this felt like abusing the patient; on the other hand, this was the only solution since they wanted patients to receive better pain relief, which was better in the long run. As one participant explained:

it doesn't feel right, ethically speaking, to hold someone down, because it feels like it's an abuse to the person who doesn't understand why you're being held down (P 9)

##### Relieve Pain and Avoid Side-Effects

The participants said that it was frustrating to wait for an FNB, knowing that one type of pain relief is superior to another.

It's very frustrating, the times when it can go many hours, it can go a whole work shift. Sometimes it isn't good in the evening and weekends before the anaesthesiologist comes and gives the FNB. It is very frustrating because I know that patients feel so incredibly much better afterward, just those with cognitive impairment to get rid of the pain, and one moment later the anxiety disappeared (P 2).

Participants also expressed how the waiting time for an FNB forced them to give analgesics, which they knew could cause unwanted side effects.

They described how it felt to weigh evil against good when waiting for an FNB. On the one hand, they wanted to give opioids to relieve patients’ pain; on the other, they knew it could cause severe side effects.

When they received the block, it is easier to communicate with the patient, they do not become as dizzy, they are easier to work with (P 14)

Some participants described how they waited as long as possible for the FNB without giving opioids, resulting in patients remaining in pain. Some reflected on how they weighed good against evil, namely how long could they allow patients to be in pain while waiting for the anesthesiologist or whether it was just better to give them opioids.

It is very nice that they are not in pain, but it becomes many other things to have control over. At the same time, you do not want to drug them either because it takes so long before they come back (P 16)

Another reason was that it was difficult to decide how far you could go to reduce the pain among patients with dementia because the groin area where the block is given is associated with intimate parts. It meant that staff needed to pull down trousers and underwear to give the block, which staff found difficult for some patients, both those with and without dementia.

I think you can inform the patient and say, unfortunately, you have to be without panties because I have to access this area, and many patients will just pull the blanket over and do not want to show anything at all (P 16).

## Discussion

Participants described their experiences of nursing care of older patients with a hip fracture who received an FNB in terms of the FNB setting the agenda. Nursing care requires timing, with staff needing to orient time and customize their communication. The outcome of the FNB affected nursing care, depending on whether the FNB was successful or unsuccessful. Further, staff faced ethical challenges regarding doing good and not harm, relieving pain, and avoiding side effects.

The meetings are short when the patient is cared for in the preoperative phase. In this study, participants expressed they needed to customize the communication because it differed among patients concerning how well they understood the aim of the block and the procedure around it, but also because only a little was required for patients to be calmer. Further, participants explained that it differed depending on which anesthesiologist performed the FNB, as some did it quickly and had no time to inform the patient about the block. Further, the effect of the FNB was described as dependent on how the anesthesiologist communicated with the patient.

Previous research showed the importance of seeing the patient in the first meeting to establish feelings of security (Hestdal & Skorpen, 2020). A review by Chan et al. (2012) found that patients have different needs and feelings regarding preoperative communication with healthcare professionals. Adequate knowledge, abilities, and positive attitudes from staff were stated as important to helping patients through the preoperative phase. A study by Miller et al. (2017) describes nurses communicating about pain and pain management as implicating challenges and dilemmas. Communication is crucial when building trust and comfort in nursing, and it can be considered the basis of the nurse–patient relationship (Afriyie, 2020). However, it is a complex, multidimensional, and multifactorial concept. When looking at the nurse–patient relationship, the nurse should be aware of the patient's perspective and previous experiences and concerns when communicating with the patient. An individual approach, competence, and a professional and ethical approach are essential communication elements (Afriyie, 2020).

Participants in this study described that when the FNB was successfully given, it was possible to do a better job, where all nursing actions became more manageable. They said that waiting a long time for an FNB increased the risk of complications. Previous research shows that patients with hip fractures are challenging to care for because they are often older and frail with multiple comorbidities. Moreover, they are in pain during the preoperative phase, making it challenging to relieve pain effectively without increasing side effects (Cowan et al., 2017; Elsevier & Cannada, 2020; Meyer et al., 2021). Patients with hip fractures need support from staff for ADL and preparations for surgery. Many may also suffer from preoperative delirium, and up to 50% have a dementia diagnosis (Unneby et al., 2020; Karlsson et al., 2020). During the preoperative phase, preparations such as a surgery shower must be made before surgery. A study from Unneby et al. (2022) showed that some patients experienced the surgery shower to be the worst part, while a randomized controlled study did not find any significant differences in preoperative delirium when comparing FNB and opioids (Unneby et al., 2020). However, in the present study, participants highlighted how patients suffered less delirium when receiving an FNB instead of opioids. Similar results were found in Henningsen et al. (2018), where patients described the value of retaining their mental awareness.

In the present study, staff faced ethical challenges regarding doing good and not harmful as they reasoned how far they could go to help the patients receive the FNB. Further, they needed to assess whether they should give opioids to relieve pain, knowing it could cause patients to suffer side effects. Haddad and Geiger (2022) point out that ethical values are fundamental to healthcare professionals. Patients have the right to decide on their beliefs and values, which can conflict with guidelines or what healthcare professionals believe is best. Healthcare professionals must minimize harm and do good to patients (Haddad & Geiger, 2022). Our results are strengthened by previous research, which states that nurses face ethical dilemmas when caring for patients. However, these dilemmas can conflict with the Code of Ethics or nurses’ ethical dilemmas and lead to moral distress (Haddad & Geiger, 2022; Rainer et al., 2018).

Participants described how they felt it was necessary to be more present with the patient after the block was given and that it became stressful since they had no time for that. Some participants reasoned whether it was better to avoid an FNB and allow patients to be in pain if it made patients try to get out of bed since staff experienced a lack of time to maintain patient safety. In another study, nurses described how it was complex to care for patients with dementia and how they lacked time to perform care in an acute care setting. However, when nurses had gathered knowledge, competence, and clinical experience regarding caring for patients with dementia, a feeling of personal satisfaction appeared (Jensen et al., 2019). Pain can cause complications and worsen the possibility of mobilization (Scurrah et al., 2018). Therefore, patients should not be excluded from receiving an FNB due to the staff's lack of time. It is more about creating organizational conditions for staff to remain present after the block if necessary.

### Strengths and Limitations

Some strengths and limitations need to be addressed. The interviews were conducted among colleagues, which can be challenging since the participants may feel uncomfortable discussing their experiences if they are consistent with the researcher or answer only what they think the researcher wants. The participants were informed that all answers were important as we tried to map various experiences. The dual roles as a researcher and a colleague must be acknowledged. A purposive sample was used to recruit participants, which is suitable when the researcher wants informants with the best knowledge concerning the research area (McDermid et al., 2014). The interviews in the present study were conducted at the same hospital, which may affect the transferability to other settings. However, to facilitate transferability, we have tried to give a clear and sufficient description of the context, selection and characteristics of participants, data collection, analysis process, and results, as well as discuss strengths and limitations (Graneheim & Lundman, 2004; Elo et al., 2014; Graneheim et al., 2017). There was only one man among the participants, which can be considered a limitation. However, women are overrepresented in care professions, where 88% of nurses are women, which makes the distribution of participants in this study quite representative (Andersson, 2019). The time to collect data can be considered long since the interviews were performed for two years. During this time, patients started, in some cases, to receive the FNB at the ED; therefore, a supplementary ethical application was submitted regarding permission to interview staff in the ED with experience in nursing care among patients with hip fractures who received an FNB.

The first and second authors are RN with long clinical experience working in an OW, and the first author has a position as an RN specializing in pain and pain management. Their preunderstandings of this context might have influenced the interviews and the data analysis process. The first authors preunderstanding can help when asking clarifying questions in appropriate places to gain a deeper understanding of the context. The results are a co-creation between researchers and participants and between the researchers and the text (Graneheim et al., 2017). The third author has no previous knowledge of OW. Working in a research group provides various understandings, different insights, and contexts, which can be an advantage. It helps prevent the researcher's preunderstandings from influencing the results, thereby strengthening the confirmability.

There are many interviews, but they can be considered relatively short. Some interviews were not rich and varied in experiences, containing more “hard facts,” but others were rich and provided more depth. Sandelowski (1995) argues that whether data are sufficient is independent of the number of participants or length of, for example, interviews. Instead, it is a matter of the richness and quality of the data. The amount of data necessary to answer a research question credibly varies depending on the complexity of the phenomena under study and the quality of data (Graneheim & Lundman, 2004; Graneheim et al., 2017). However, it requires that the researchers can analyse and simplify the data and form categories that reliably reflect the study topics to maintain a successful content analysis (Graneheim et al., 2017; Elo et al., 2014).

### Implications for Practice

Staff describe their experiences of nursing care of older patients with a hip fracture who received a femoral nerve block in terms of the femoral nerve block setting the agenda. The nursing care was affected by the femoral nerve block and depended on if the femoral nerve block was successful or not. Thus, the femoral nerve block should be the base of pain management guidelines and be administered as soon as possible to facilitate nursing care among patients with hip fractures

## Conclusions

The femoral nerve block was an important issue for nursing staff in patients with hip fractures in the preoperative phase. Nursing care requires timing and the outcome of the FNB depends on whether the FNB was successful or unsuccessful and thus affected nursing care. Our results point towards the benefits of giving FNBs as soon as possible to facilitate nursing care; however, this should be studied in future research.
